# LIFE HISTORIES AND TRAJECTORIES OF PHYSIOLOGICAL FUNCTIONING AMONG OLDER RACIALIZED GROUPS IN THE US

**DOI:** 10.1093/geroni/igae098.1365

**Published:** 2024-12-31

**Authors:** Catherine García, Morgan Parella, ’Lisia De Adorno, Eniola Festus, Londi Rowell

**Affiliations:** Syracuse University, Syracuse, New York, United States; Syracuse University, Syracuse, New York, United States; Syracuse University, Syracuse, New York, United States; Syracuse University, Syracuse, New York, United States; Syracuse University, Syracuse, New York, United States

## Abstract

Enhancing later life health outcomes among racialized groups demands a nuanced exploration of early life and adulthood circumstances, recognizing their profound impact on individual life trajectories and health disparities. This study used a subsample of age-eligible non-proxy participants from the 2006-2020 biennial waves of the Health and Retirement Study (HRS) that identified as White Hispanic/Latinx (n=787), non-White Hispanic/Latinx (n=516), non-Hispanic Black (n=2072), and non-Hispanic White (n=7984). We conducted a group-based latent class analysis to characterize distinct types of life course experiences among middle-aged and older adults, including childhood family context and residence, early-life educational experiences, and health before age 50. Then, we examined the associations between identified life course typologies and trajectories of physiological functioning using linear mixed models. All independent variables and covariates were interacted with age to discern their influence on the rate of change in physiological functioning. A 4-class model was fit for childhood family context and residence, early-life educational experiences, and health before age 50 by race and ethnicity that exhibited varying patterns of (dis)advantaged life course experiences. The parameter estimates for physiological functioning show that non-Hispanic Black and non-White Hispanic/Latinxs in the class characterized with more disadvantaged experiences exhibited higher levels of poor physiological functioning at baseline and over time relative to their more advantaged counterparts. This analysis offers a novel perspective on the intricate interplay between life course experiences and health trajectories, shedding light on critical junctures for targeted interventions to improve the health and well-being of racialized groups in midlife and beyond.

